# Cocaine and Levamisole Induced Vasculitis

**DOI:** 10.7759/cureus.17192

**Published:** 2021-08-15

**Authors:** Harpreet Gill, Dylan Trinh, Danyon J Anderson, Nathan Li, Devin Madenberg

**Affiliations:** 1 Hospital Medicine, Medical College of Wisconsin, Wauwatosa, USA; 2 School of Medicine, Medical College of Wisconsin, Wauwatosa, USA; 3 Internal Medicine, Medical College of Wisconsin, Wauwatosa, USA

**Keywords:** levamisole-induced vasculitis, cocaine levamisole-induced vascuiltis, anca-associated vasculitis, levamisole, cocaine

## Abstract

Levamisole adulterated cocaine is a rare cause of anti-neutrophil cytoplasmic antibody (ANCA) associated vasculitis. It is increasingly diagnosed because of raised awareness; however, it is still underdiagnosed in part because of its rarity and patients not reporting cocaine use. Here we report a case of levamisole-induced vasculitis. We present a 48-year-old non-Hispanic white male with a past medical history significant for Crohn’s Disease and pneumonia who presented with acute bilateral ear pain and rash. His urinary drug screen was positive, which prompted suspicion of contamination and potential levamisole adulterated cocaine-associated vasculitis. A punch biopsy showed evidence of leukocytoclastic vasculitis and multiple fibrin thrombi further supporting contamination with levamisole. We believe this case highlights the importance of using patient history in guiding diagnostic testing in the setting of acute vasculitis. Once the history of illicit substance use was confirmed, our differential diagnosis and considerations for treatment significantly changed.

## Introduction

Cutaneous vasculitis can be caused by several distinct etiologies, including cutaneous lupus, meningococcal sepsis, cutaneous squamous cell carcinoma, graft-versus-host disease, thrombotic disorders, angiosarcoma, embolic disease, hematologic malignancies, paraproteinemias, and Stevens-Johnson Syndrome [1]. Here, we present a case of anti-neutrophil cytoplasmic antibody vasculitis (AAV) following cocaine use, which we attribute to Levamisole adulterated cocaine-associated vasculitis. In a 20-year ecological study published in 2017, the incidence of AAV was found to be 1.2-2.0 cases per 100,000 individuals and a prevalence of 4.6-18.4 cases per 100,000 individuals [2]. 

Through a national survey conducted in 2009, it is suspected that Levamisole contaminates approximately 70% of cocaine in the US [3], with more recent reports approximating this to be higher than 80% [4]. Due to the high number of distinct etiologies of acute vasculitis and likely increased incidence of this syndrome, patients with suspected Levamisole adulterated cocaine-associated vasculitis require extensive workup to verify the correct diagnosis. 

## Case presentation

**Figure 1:**
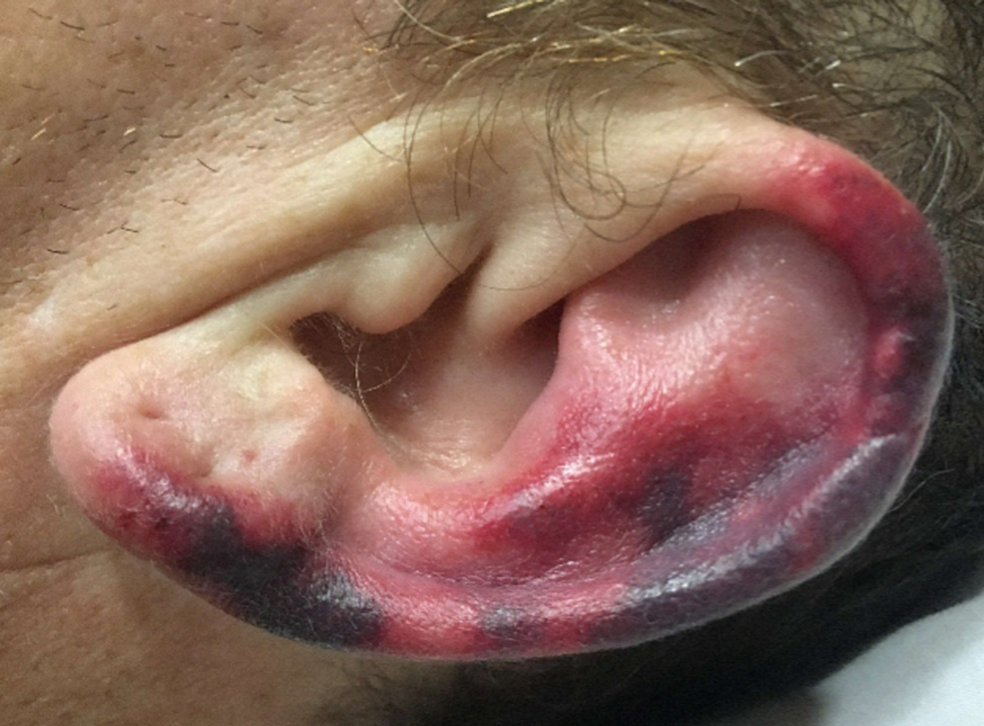
Vasculitis on Ear Rash on the left ear showed retiform purpura with few hemorrhagic bullae. No hand, foot, or other facial involvement was reported. This ear rash is a hallmark of levamisole-associated vasculitis.

**Figure 2:**
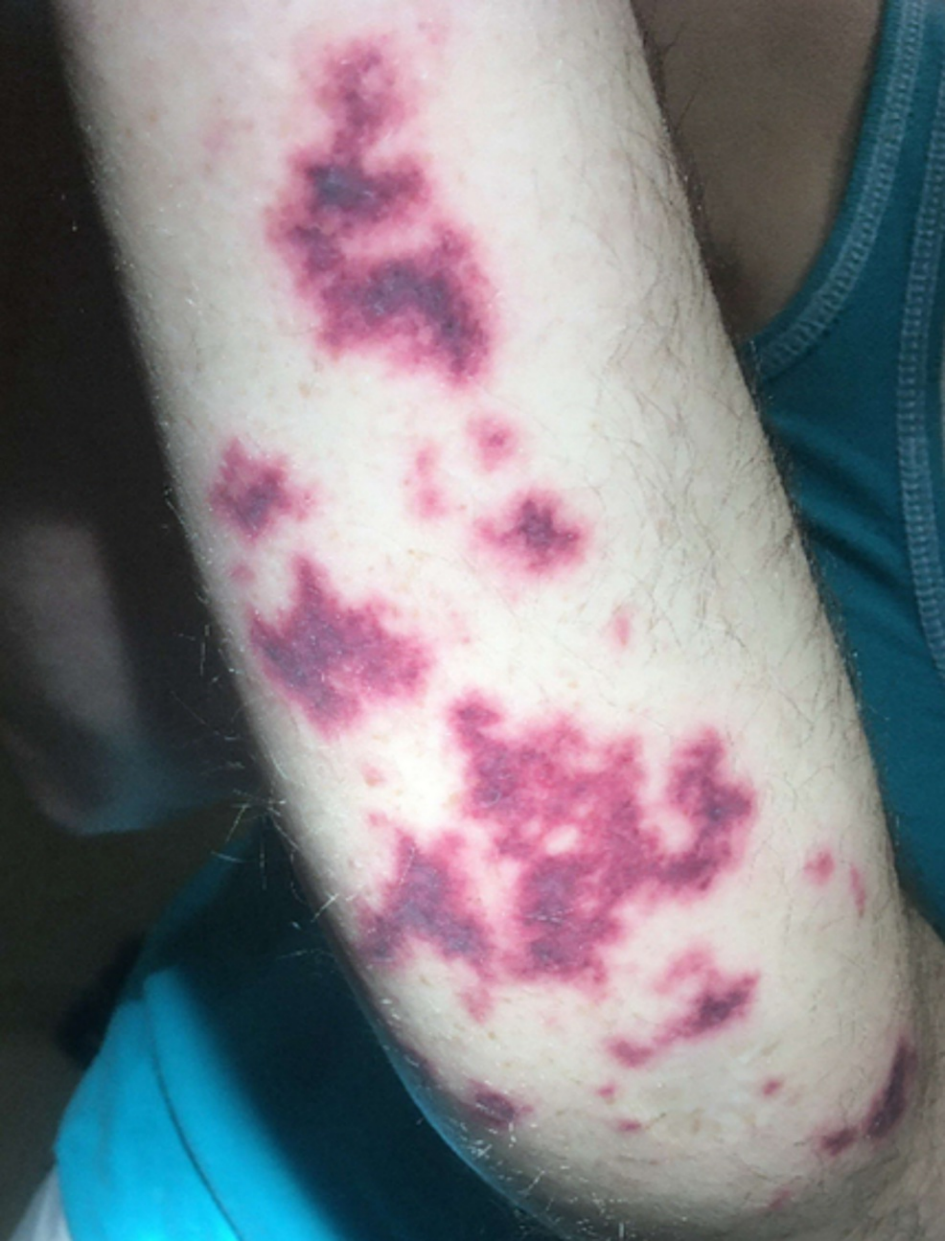
Vasculitis on Right Upper Extremity Rash on the right upper extremity, which was non-ulcerative, scattered, retiform purpura. Similar, but milder, findings were seen on the lower extremities.

A 48-year-old non-Hispanic white male with a past medical history significant for Crohn’s Disease and pneumonia presented with acute bilateral ear pain and rash. Following an initial assessment, the patient was discharged on empiric antibiotic treatment for presumed otitis media. The patient represented to care two days later with worsening purpuric and tender rash which had spread to his left upper extremity and bilateral lower extremities (Figures 1, 2). He stated he had a similar rash approximately one year prior which resolved without medical intervention. The rash also showed no signs of blanching. The leading differential diagnosis became a vasculitis of unknown etiology, possibly rheumatoid arthritis given the history of autoimmune disease, and the patient was discharged on prednisone. 

The following day, the patient returned to care with no improvement in symptoms and progressive cough. He reported that he had not used prednisone since the previous encounter. He also reported the use of crack cocaine prior to the initial onset of rash at this encounter. This was confirmed with a urinary drug screen, which prompted suspicion of contamination and potential levamisole adulterated cocaine-associated vasculitis. Serology was ordered and supported this hypothesis as both antineutrophil cytoplasmic antibodies, cytoplasmic (c-ANCA) and antinuclear antibody (ANA) were positive with values of 1:10240 and 1:160 respectively. Myeloperoxidase (MPO) was negative with a value of 17 au/ml. A punch biopsy showed evidence of leukocytoclastic vasculitis and multiple fibrin thrombi further supporting contamination with levamisole. This microscopic presentation supported levamisole-induced vasculitis and ruled out other causes of vasculitis including microscopic polyangiitis. The acute onset of this patient's presentation without associated chronic complications rules out diseases like granulomatosis with polyangiitis. Once the diagnosis was made, it became known that the patient was experiencing an acute condition and that no chronic conditions causing this vasculitis needed to be treated. Care became supportive and after the rash improved, the patient was discharged to follow up with a primary care provider to monitor recovery. 

## Discussion

Cutaneous vasculitis, while often benign, may occasionally present due to complex underlying etiology. Vasculitis is most often due to infections (such as hepatitis B or C), blood cancers, autoimmune diseases, and drug reactions [5]. It is important to identify the cause of vasculitis because treating the underlying cause is crucial for long-term treatment. For example, alpha-interferon and lamivudine are used to treat poly-arteritis nodosa by treating hepatitis B [6]. Surprisingly, the cause of vasculitis, in this case, was a common additive in cocaine. 

Impurities in cocaine, such as levamisole, have been associated with vasculitis [1]. The proposed pathophysiology is the metabolism of levamisole in the liver by aminorex, which may be detected in urine samples for up to two days after ingestion [7]. Aminorex is an amphetamine-like substance that potentiates cocaine activity via increased release of catecholamines, most prominently norepinephrine, dopamine, and serotonin, leading to vasoconstriction and vascular remodeling due to its prolonged half-life [4]. Clinical presentation of levamisole contaminated cocaine-induced vasculitis is most commonly purpura. Diagnosis is made primarily upon histology and positive perinuclear anti-neutrophil cytoplasmic antibodies (p-ANCA), MPO, and ANA result upon serology following a positive urinary screen for cocaine use. Typically, levamisole adulterated cocaine-induced vasculitis resolves spontaneously, and thus treatment is most commonly supportive care, though steroids may be prescribed as needed. 

Interestingly, levamisole was used as a treatment for inflammatory bowel disease (IBD) and was associated with side effects including arthritis [8]. The association between levamisole vasculitis and IBD remains unexplored in the literature, but it is possible that having IBD increases the risk of having levamisole-induced vasculitis.

In this case, once the etiology of the vasculitis was determined, supportive care was sufficient to manage the rash. We believe this case highlights the importance of using patient history in guiding diagnostic testing in the setting of acute vasculitis. Once the history of illicit substance use was confirmed, our differential diagnosis and considerations for treatment significantly changed. 

## Conclusions

Levamisole is the most common cocaine additive and may cause multiple immunologic presentations including vasculitis. Social history, in particular illicit substance use, should be considered in AAV presentation, especially with retiform purpura in the ear. Lab results that are typical for levamisole-induced vasculitis are positive p-ANCA, MPO, and ANA results upon serology following a positive urinary screen for cocaine use. Levamisole vasculitis spontaneously resolves, so supportive treatment and steroids are routine. In this case, supportive care was a successful treatment. This case emphasizes the usefulness of patient history in guiding the diagnosis of vasculitis. In this case, the patient's reluctantly given the history of illicit substance guided our diagnosis and treatment. 
